# Civil society and medical product access in Africa: Lessons from COVID-19

**DOI:** 10.3389/fmedt.2023.1091425

**Published:** 2023-02-07

**Authors:** Janet L. Wale, Kawaldip Sehmi, Regina Kamoga, Robert Ssekubugu

**Affiliations:** ^1^ Independent Researcher, Brunswick, VIC, Australia; ^2^ International Alliance of Patients' Organisations, London, United Kingdom; ^3^ Uganda Alliance of Patients Organizations (UAPO), World Patient Alliance (WPA), CHAIN, Kampala, Uganda; ^4^ Rakai Health Sciences Program Research Institute in Kalisizo, Kalisizo, Uganda

**Keywords:** civil society engagement and participation, regulatory lifecycle and value assessment, human rights, social cohesion, low and middle-income African countries

## Abstract

Understanding health as a human right creates a legal obligation on countries to ensure access to timely, acceptable, and affordable health care. We highlight the importance of a meaningful role for civil society in improving access to well-regulated quality medical products in Africa; to support and be part of a regional social contract approach following the access issues that have been particularly evident during the COVID-19 pandemic. We argue that African communities have a clear participatory role as important stakeholders in the regulatory lifecycle. Solidarity is important for a cohesive approach as formal government healthcare infrastructure may be minimal for some countries, with little training of communities available for disease management and insufficient money to fund people to organise and deliver health care. Some of the issues for civil society engagement with multi-stakeholders, and possible mitigating strategies, are tabulated to initiate discussion on facilitators and concerns of governments and other stakeholders for meaningful participation by patients, communities and civil society within a regional regulatory lifecycle approach. Solidarity is called for to address issues of equity, ethics and morality, stigmatisation and mutual empowerment – to sustainably support the region and national governments to develop greater self-sufficiency throughout the regulatory lifecycle. By creating a participatory space, patients, communities and civil society can be invited in with clear missions and supported by well-defined guidance to create a true sense of solidarity and social cohesion. Strong leadership coupled with the political will to share responsibilities in all aspects of this work is key.

## Definition

Civil society: Communities and groups that work outside of government or commercial bodies. The sector of society distinct from government and business, and including the family and home.

## Introduction

Understanding health as a human right creates a legal obligation on countries to ensure access to timely, acceptable, and affordable health care of appropriate quality. This is in addition to providing for the underlying determinants of health such as safe and potable water, sanitation, food, housing, health-related information and education, and gender equality (1). A rights-based approach to health requires that health policy and programmes must prioritise the needs of those furthest behind first, towards greater equity, without discrimination on the grounds of race, age, ethnicity or any other status. Another important feature of rights-based approaches is active participation. That national stakeholders are meaningfully involved in all phases of assessment, analysis, planning, implementation, monitoring and evaluation. This includes community and non-state stakeholders such as non-governmental and civil society organisations (1, 2). Community and patient experiences, burdens of disease, patient needs and issues of equity and stigma are important considerations in health care (3), where value assessments are influenced by social and historical settings (4). Some countries may however lack political freedom or will to transparently discuss and define their priorities in building the required resources to deliver on health care.

### An existing model

The United States. President's Emergency Plan for AIDS Relief (PEPFAR) is an example of a United States. government's global effort to change the trajectory of the global HIV epidemic. This program has sought to move to a model of country ownership (governments and organisations), not just local non-government organisations (NGOs) (5). Sustainable global health programs therefore ultimately require states to mobilise resources and channel funding to directly manage such programs.

The objectives of the present project are to:

1) highlight the importance of human rights in equitable access to health care and medical products in Africa, and the active participation of patients, communities and civil society in policies and strategies to build effective and cohesive regulation, value assessment and appropriate actions within the regulatory lifecycle;
2) identify some of the challenges to providing quality medical products in Africa;
3) Identify issues for patient, community and civil participation with possible mitigating strategies, to enable meaningful engagement.

Our intention is that through this work we can raise awareness, inform discussions, trigger actions and promote further study. An empirical review of the current literature was applied to inform the project. We also searched selected grey literature sources, collated sources already known to the authors and put out requests to our networks (including through social media). When we identified papers, we checked the references of those papers.

### Access to quality medical products in Africa – the developing situation

The world's experience of the COVID-19 pandemic has made it clear that Africa as a region cannot solely rely on charity from higher income countries to provide essential health care to meet therapeutic needs in line with International Human Rights (6–8). African countries need to work together within a social contract model (9) to build relevant healthcare infrastructure, capacity and medical supplies for the health security of their individual populations. The region needs to strengthen its own pharmaceutical industry to manufacture vaccines and other essential medical products to improve supply and access (10).

The existing Africa Centres for Disease Control and Prevention (CDC), and its regional collaborating centres, was officially launched in January 2017 and is a public health agency of the African Union (AU). It was set up to support the public health initiatives of member states by strengthening capacity and capability of Africa's public health institutions based on data-driven interventions and programmes (11). Overall, it is recognised that the region would be well-served by coordinating and collaborating its efforts, communicating clear missions, guidance and evaluation strategies, and demonstrating solidarity between countries to increase negotiation power and access to medical products. During COVID-19, efforts to obtain the vaccine were hindered by protective intellectual property rights provided through patents on technologies, know-how, manufacturing processes and other trade secrets (12, 13). The AU has worked hard over recent years to initiate the African Medicines Agency (AMA) for regulation and approval of medical products (14). An African Pharmaceutical Technology Foundation has been set up to establish technologies that are important for the manufacture of products (10). The AMA was ratified in November 2021 and its Secretariat is being set up in Rwanda (15, 16) as is that of the Technology Foundation. Low income countries are known to suffer from diseases that attract little investment by the global pharmaceutical industry. Preventative treatments could also be more affordable and their uptake increased, as demonstrated for pre-exposure prophylaxis against HIV in the United States (17). Responsible innovation in health is indeed an issue in high income countries leading to calls for collaborative efforts to clarify and set ethical, economic, social and environmental principles, values and requirements to design, finance, produce, distribute, use or discard socio-technical challenges and possible solutions (18, 19). Worldwide, changes are needed to build sustainable, participatory health systems that meet genuine therapeutic needs.

### Identified needs and barriers

Early in the COVID-19 pandemic, it was estimated that the risk of dying from the disease was roughly twice as high for people living in lower-income countries as for those in rich nations. By the end of 2021, 64.1% of people living in high-income countries had received at least one dose of COVID-19 vaccine compared to only 5.4% in low-income countries (20). The marketing and political power of a few global vaccine manufacturers were under the spotlight in a situation that totally neglected health-equity principles (21). Nationalism and hoarding kept technological developments for COVID-19 within high income countries (17, 22). Licensing agreements for the manufacture of vaccines in low and middle income countries (LMIC) could not be reached. Refusal to license and transfer the vaccine technology meant that the World Trade Organization Trade Related Intellectual Property Rights (TRIPs) perpetuated inequitable access to COVID-19 vaccines (23). Stimulating and rewarding innovation is one of the main purposes of patents, together with data and market exclusivity, and large parts of the world were left unprotected from the pandemic even though this could lead to the rise of new variants. Social cohesiveness and solidarity within populations meant that some countries did relatively well during the early phases of the COVID-19 pandemic, for example Japan, Taiwan, Vietnam (24). This showed that if all individuals are considered fairly and equitably in a socially cohesive system then challenges may be addressed in a rational way. Cohesiveness could also contribute to better monitoring and data collection to inform decision making based on epidemiological data and subsequently rapid and efficient control of epidemics.

### Particular needs of African countries for access to safe and effective medical products

Africa has a heterogeneous population made up of different cultures and beliefs (25, 26). Geographic location, weather, transport, and other logistics together with procurement, limited infrastructure and staffing strongly influence access to health care in the region (27, 28). The International Declaration of Human Rights (1) is a primary concern with regard to access to health care and medical products, where civil society and community groups have important roles to play (27, 29). Difficulties with procurement, distribution, and storage, particularly when electrical supply, refrigeration and cold storage are needed, continue to limit access to medical products (27).

An important role of regulatory bodies is to enable processes to determine and ensure the safety, documentation, quality and performance of medical products, including medicines and vaccines, medical devices and technologies. These processes determine that the product is effective when compared with placebo or usual treatment and is safe for marketing and access within a population (30). Involving civil society in decision making can increase the quality of the decisions and ensure that new therapies address the specific needs of local communities (31, 32). Not all African countries have regulatory systems that can effectively manage safe entry of medical products into their countries, or enable their manufacture. A Global Benchmarking Tool (GBT) is used by the World Health Organization (30) to evaluate national regulatory systems. The GBT identifies strengths together with areas for improvement and ways to address gaps. This allows assessment of the overall ‘maturity’ of the regulatory system with Rwanda and some other African countries (eg Ghana, Nigeria, and Tanzania) reaching targets (30).

By working together at a regional level, the more highly (M3) qualified systems can support other countries in developing effective regulatory processes to control the quality and availability of medical products, by whatever mechanism they enter the region (16). Yet working toward an evidence informed system that is transparent, participatory and consistent has been identified as costly and requires expertise, institutional capacity, funding and time (33). These elements can be in short supply in LMICs, where analytic and administrative capacity is limited, funding and human resources are scarce, and governments may be hesitant to restrict their own discretionary powers (34).

### The role for civil society in African subregional systems

We set out to demonstrate the importance of a meaningful role for civil society in going forward in Africa. The AU is working with multiple partners including the European Medicines Agency (EMA), national regulators in Europe and elsewhere, and funding partners to leverage international experiences in moving forward with the AMA (16). The African Pharmaceutical Technology Foundation is being set up under the auspices of the African Development Bank to promote and broker alliances between foreign and African pharmaceutical companies and others to build collaboration between the public and private sectors, for example African Union Commission, European Union Commission, WHO, the World Trade Organization, Medicines Patent Pool (MPP) and other philanthropic organisations, bilateral and multilateral agencies and institutions (10). We argue that local communities are important stakeholders in access to medical products and have a clear participatory role in the medical product lifecycle in Africa. Drivers for participation include equality and equity, dealing with stigmatisation, ethics and morality, and the need to sustainably support national governments to deliver effective health services. Discussion is needed on facilitators for patient, civil society and community engagement and ways in which the concerns of governments and other stakeholders can be addressed.

### Human rights and social cohesion

The WHO sees that the purpose of healthcare innovation as to deliver new and improved health policies, systems, products and technologies, services and delivery methods (35). The United Nations (UN) has set a goal for Universal Health Coverage (UHC) in LMICs as part of the 2030 Agenda for Sustainable Development (36). The WHO took up the challenge of equitable access to core essential medicines in all countries of the world by developing a regularly updated, evidence-based essential medicines list (EML) to focus activities including manufacture at a country level (37). The EML can, however, be seen as limiting the availability of medical products for some people where treatments may not be on the list causing individual countries to be challenged to look at how they can extend their EMLs to new potentially effective treatments, for example in Thailand (38). This is particularly so for people with disabling and life-threatening diseases, such as cancers and rare diseases, where people become aware of the new treatments through international patient networks (eg 39, 40), industry alerts and access schemes (eg 41, 42). Human rights can be seen to offer an important mechanism for citizens to petition for additional government resources and for delivery on health services considered high priority. International Human Rights law demands the fulfilment of ‘core obligations’ by states including for national strategies across entire populations with plans of action based on burden of disease and through a legitimate and participatory process (43). As an example, people living with a rare disease are at greater risk of stigmatisation and discrimination, creating obstacles to their full participation in society. The United Nations General Assembly in 2021 adopted a Resolution on ‘Addressing the challenges of persons living with a rare disease and their families’ to promote and protect the rights of everyone living with a rare disease (44).

### Risk management, actions

As part of the regulatory lifecycle, health technology assessment (HTA) is used by a country or region to determine the value of a medical product within its health system (26, 32, 38, 45). HTA with its multi-stakeholder involvement can inform decision making on reimbursement and universal health coverage (UHC) in a way that can be used to resolve policy issues, including right to rescue arguments (46). Incorporating evidence from the disciplines of social and behavioural sciences can enrich the regulatory lifecycle approach and enhance the value of the evidence to inform policy (4). HTA can also bring greater monetary benefit compared with a first come, first served approach, as shown for Thailand (47). Regulatory methodologies continue to evolve and it has become apparent during the Covid-19 pandemic that regulatory and HTA bodies can be more effective if they work together, with clinicians and civil society involved throughout (48, 49). There is a good opportunity in LMICs, where capacity is needed, to design purpose-built systems (49, 50). Recent publications have highlighted the important role of patient and public involvement in economic modelling for HTA in the United States (51–53). Patients and civil society can bring their experiential knowledge into model development and evaluation of the clinical safety and efficacy and the quality of the evidence as part of the economic assessment where more complete effectiveness is a prime target.

### Medical product supply

Medical products are generally developed for market by industry, and sold as national private goods. We have used the term medical product to be inclusive of innovative technologies under development, including diagnostics and medical devices. Overall, the ‘supply’ side (innovation policy-makers, entrepreneurs, investors) in high income countries does not align with the ‘demand’ side (health policy-makers, regulators, value assessors/health economists, payers) and the needs of patients or community. And clinical trial data is also not shared transparently or well (54) in an environment where ‘value’ means different things to each of the key players in a health system. Responsible innovation principles would address societal challenges in alignment with the objectives of UHC while enabling health innovation (4), where moral, political and power relationships come into play in determining access and use of new technologies (18).

### Innovation in medical product supply

Large, global pharmaceutical companies have changed their business models over recent decades. Companies may no longer operate their own drug-directed research laboratories but buy in scientific and technological advances. Early-stage medical products or ideas can come from publically funded research in universities and public research institutions as well as small enterprises (19b, 22), for example much of the foundational work on mRNA vaccines was conducted at universities over many years. This situation has led to the concept of socially responsible licences for inventions emerging from public research, where companies could then use their infrastructure, skills and expertise in developing scientific advances into medical products for use in health systems.

Furthermore, the boundary between basic biomedical research and clinical studies on treatments for disease has narrowed, where seriously ill patients are keen to seek the latest scientific developments in an attempt to extend their lives. As the complexity of the medical products and their development increases so does the cost of medical products, making them less accessible to those in need of treatment, and challenging the sustainability of health services (55, 19). In closed, regulated markets, it is important that medical products are assessed for safety and performance, and to determine their value and benefits to a healthcare system constrained by a finite health budget. Innovation is generally associated with profitable business models where it is predicted the product will have health benefits for a particular disease area. Pharmaceutical companies have an obligation to their shareholders to increase their markets. They do not have to ensure that technologies entering the market are either desirable or cost-effective (56, 57) such that not all innovative medical products or technologies add value nor are sustainable for healthcare systems. It is to their benefit to be the first company to bring a new medicine type to market, and so they reap economic value from the regulatory process (56). Other companies develop similar products, eventually leading to many products in the same therapeutic area. Marketing skills, how the drug is delivered and side effect profiles as well as costs, play a role in how the market evolves. Confidentiality of data on the new medical product is therefore important. On the other hand, the pharmaceutical industry has responsibilities to respect human rights, and to be held accountable (2, 58).

### Corporate responsibilities

The Fair Pharma Scorecard is a project of the Dutch-based non-profit organisation Pharmaceutical Accountability Foundation (PAF) that takes action against unreasonably priced medicines and abuse of market exclusivity rights to keep prices high. The scorecard ranks pharmaceutical companies on how well their policies and practices reflected a commitment to human rights principles during COVID-19 (59). The information is used to inform the public and take legal action, if necessary.

Collecting patient and community experience data is key to person-centred health care and medical product and technology development. Industry, regulators, research foundations, patients and communities are actively working to improve the use of meaningful patient experience data in medicines development in the US, Europe, and more widely (60, 61). The Council for International Organizations of Medical Sciences (CIOMS) is an international, non-governmental, non-profit organization with the mission to advance public health through guidance on health research and policy including ethics, medical product development and pharmacovigilance. It has released a report on systematic patient involvement in the development, regulation and safe use of medicines, incorporating views gathered from an open multi-stakeholder international meeting in Switzerland and a workshop in Uganda (61).

An increasing number of pharmaceutical companies are setting up schemes to provide their products to low-income countries on a ‘not-for-profit’ basis. These companies include GSK, for infectious diseases (62), Sanofi in therapeutic areas such as diabetes, cardiovascular disease, tuberculosis, malaria and cancer (63) and Pfizer covers medicines and vaccines that treat infectious diseases, certain cancers and rare and inflammatory diseases (64). The latter states that it will work with countries “to identify quick and efficient regulatory pathways and procurement processes to reduce the longer amount of time it can take to make new medicines and vaccines available”. It is aligned with the Bill & Melinda Gates Foundation for new vaccines (64). After providing access schemes for new medicines, companies then consider it is up to governments to provide the medications to patients in the longer term (64).

### Stakeholder engagement, responsiveness, social value and the needs of civil society

HTA appraisal of the evidence on medical products or technologies and therefore their value is conducted by interdisciplinary multi-stakeholder groups, often using explicit analytical frameworks (65). Engagement with relevant stakeholders, with clear roles and responsibilities, can ensure ownership of the regulatory lifecycle approach. The level of buy-in from each stakeholder, including patients, is crucial for successful implementation of decisions and offers a clear mechanism to look at country or region specific needs and values (66). A ‘window of opportunity’ is provided to gain political and public support for evidence-informed decision making using legitimate, well-defined evidence-informed processes that are legally defensible (67). This requires engaging patient communities early and in a gender-sensitive, ethical, culture-appropriate and sustainable way (25). An important hurdle to overcome is a lack of trust between the relevant stakeholders (policy makers, administrators, researchers, industry, clinicians, civil society including patients and communities) and to recognise and address inherent vested interests (50). The WHO 2021 Manual for UHC provides guidance on community engagement, starting with formalising structures to create a safe space established within a legal framework. A common under­standing among relevant stakeholders is needed of what the participatory space is and will be, with functional guidance on roles and responsibilities (29). The need for mutual respect is of prime importance, allowing trust in the processes to develop over time. Community benefits of participation need to be evident and with a clear understanding of roles and implementation of the decisions to be made.

Some of the issues to be addressed on patient and community engagement are given in Table 1, together with possible ways of mitigating them. The table is a collation of the literature and experiential expertise, drawn together by the authors. A working version was shared for comments to known patient advocate leaders with good knowledge of HTA. We have not stratified the issues based on impact or risk as we see that as part of future work, when the context has been established.

**Table 1:** Collated identified issues for meaningful civil society engagement with multi-stakeholders, and possible mitigating strategies, based on the literature and experiential knowledge.

Issues for civil society engagement	Mitigating strategies
The need for mutual respect, and trust	Must be earned, maintained over time
Communities may not be organised eg into patient and disease-based groups or organisations	Incorporate Real World Evidence and therefore patient experiences (surveys, PROMs, PREMs, patient preference studies). The need to use a range of ways to seek the input and views of diverse patients, carers, families and communities
Economic, educational and power inequalities that can cause distrust	May best be worked on over time by initially involving capable individuals who understand the issues and have a strong connection to patients and civil society
Lack of funding: little or no money, or with budget restraints and the need to support a community in their care	Make a part of usual business to collect information/data on experiences
Lack of understanding of role and responsibilities, processes	Education and training packages, one-to-one conversations, mentoring, train-the-trainer programs
Some communities may have unrealistic expectations of what benefits or compensation and support the community and community partners may receive	Provide transparency about how money is spent and working goals; sharing the concept of a social contract, and longer term goals
Demands from communities—to address the burden of disease that is unrelated to the area of immediate deliberation. For example, factors such as poverty, malnutrition, low (health) literacy, lack of healthcare infrastructure and high disease burden	Slowly develop trust (ideally within a social contract) that people are working toward principles of greater transparency and documented sharing of information and evidence from data collection
A concern that disease-based support groups place their own treatment needs above the needs of other patients	Develop trust (ideally within a social contract) that people are working toward well documented and transparent prioritisation processes
Ethical issues such as any risks of participation or any form of harm, role of gender in representing communities	Working sustainably over time, with good shared documentation of efforts, to equitably meet the needs of the entire community, with cultural awareness
Lack of education, knowledge, training and capacity to be able to engage, targeted at patient/civil society groups, community, individuals	Share good practices
Lack or ‘representativeness’, particularly within mixed/heterogeneous communities	Encourage organised groups to seek wider perspectives and viewpoints, to report back on
If there any community benefits of participation	Increased understanding of the decisions to be made, and how they are made
Leakage of clinical trial data and information – As each company tries to keep its early trial data as confidential as possible eg in delaying release of clinical trial data and the content of its submissions. This could potentially reduce the competitive edge for a company	Upskilling on need for confidentiality with the knowledge that stakeholders are respectfully working to achieve the goals of a participatory approach to decision making in health care
Engaging patient groups with financial relationships with industry, which might lead to conflict of interests and subsequent potential threats to the integrity and independence of the stakeholder groups	Transparency about corporate funding. Need to build on transparency about corporate funding – need to address in whatever way can, and with all stakeholders so all potential conflicts become evident
‘Expressiveness’ is intertwined in one's culture, experiences, values, and self. Need to create the participatory space and invite patients, communities and civil society in	Provide space for the many voices, faces, and mannerisms
Takes resources and effort	Upskill, monitor and evaluate – to enable continuous improvement

### A participatory approach

Rights are a source of power if enabled. A participatory space for patients, communities and civil society would bring power to those who have less of it, and for those whose voices are generally weaker and whose health is often poorer (29). A culture of engagement and participation is most effective when backed by legislation affirming the right to health and participation and providing a legal framework to build capacities in fair and transpar­ent participatory processes. The patient, community and civil society population would benefit by being instilled with a sense of social cohesion and duty to participate, to achieve recognition of their roles with meaningful outcomes. A culture of participation relies on development of trust and respectful relationships, across all participants. A provision which is often left out of participatory models is the need to build the capacity to enter, engage with, and maintain a participatory space for communities and civil society organisations – enabling requisite training, coaching, and supervision (29). The level of grassroots and civil society activity evident today provides an indication that the time is ripe for legislation to provide a ‘participatory space’. We need to capitalise on the capacity and experience of those who are already active, and take into account their knowledge and local expertise, as with IAPO (39). The African Medicines Agency Treaty Alliance (AMATA) was set up under IAPO as a multi-stakeholder alliance to advocate for the ratification and implementation of the AMA Treaty and provide meaningful engagement with patients and other relevant parties in all aspects of its work (65). AMATA has a Steering Committee that comprises of representatives from its members, patient groups and civil society organisations, NGOs, industry associations, research and academic networks, youth and advocacy groups (68). Another example is the World Patients Alliance, where patient and community groups intermingle with other such people across the globe to address patient safety (69). A civil society that is educated to world-class standards, with the ability to adopt and professionally adapt technical expertise is important in moving forward. Collaborative programs and exposure to other international bodies and study programs can help with this if aware of the unique context of the African region and the need for decolonialism.

## Contribution to the field

Low and middle-income countries in Africa did not have access to COVID-19 vaccines that were available to high income countries early in the pandemic. This has demonstrated that the Africa region cannot rely on charity to meet its preventive and therapeutic needs. International Human Rights have not protected its populations. Donated products are often of inferior quality when received, which adds to the need for strong regulation, value assessment and appropriate actions for medical products within the regulatory lifecycle. The newly formed African Medicines Agency and African Pharmaceutical Technology Foundation could have important roles. Civil society participation in health care and medical product decision making is written into International Human Rights, and country governments as well as industry have duties and responsibilities to uphold these rights. We outline concerns that have been raised in involving patients, communities and civil society as meaningfully and actively participating stakeholders within the regulatory lifecycle, and steps for building mutual respect and trust. Solidarity and social cohesion are important in moving forward to ensure that medical products are effective, safe and affordable and available to the people who need them.

## Conclusions

African countries have learned through the COVID-19 pandemic that they cannot rely solely on charity to provide essential medical products in a timely way. In line with International Human Rights, Africa needs to strengthen its own biopharmaceutical and regulatory lifecycle industries. With solidarity and social cohesiveness the region can develop its own manufacture and increase access and negotiating power for medical products and technologies. Regional change is needed to build sustainable participatory health systems that have strong regulatory lifecycle structures to meet therapeutic needs, building expertise and capacity aligned to local needs through a participatory approach that meaningfully involves patient, communities and civil society together with other key stakeholders. We have outlined some of the background issues together with concerns and ways to address them in promoting patient, community and civil society participation in regulatory lifecycle policy, value assessment and decision making.

We have a human right to health and essential medical products, but the drive for innovation has exaggerated inequities in delivery of health care and added to ever-increasing complexities and costs in a market where products developed by industry are sold as national private goods.

Collating patient and community stories and data on experiences with healthcare services and medical products is key to person-centred health care, and in medical product development and value assessment. Global biopharmaceutical companies are setting up not-for-profit schemes to provide their medical products to low-income countries. But sustainable health programs ultimately require states to take over management and funding of the programs that provide these services. Understanding health as a human right creates a legal responsibility for countries to ensure access to timely, acceptable, affordable health care of appropriate quality, in addition to addressing other social determinants of health. An important feature of human rights is meaningful participation by civil society across the regulatory lifecycle, policy and value determination in a gender-sensitive, ethical, culture appropriate and sustainable way.

Community benefits of participation may become evident with clear understanding of roles, processes and responsibilities for implementation of decision making.

## Data Availability

The original contributions presented in the study are included in the article/Supplementary Material, further inquiries can be directed to the corresponding author.

## References

[1] The International Declaration of Human Rights. Available online at: https://www.who.int/news-room/fact-sheets/detail/human-rights-and-health (accessed October 25, 2021).

[2] United Nations (UN) Guiding Principles on Business and Human Rights. Pharmaceutical industry responsibilities to respect human rights, and to be held accountable. Available online at: https://www.ohchr.org/sites/default/files/documents/publications/guidingprinciplesbusinesshr_en.pdf (accessed May 16, 2022).

[3] Van der Wilt G Bloemen B Grin J Gutierrez-Ibarluzea I Sampietro-Colom L Refolo P Integrating empirical analysis and normative inquiry in health technology assessment: the values in doing assessments of health technologies approach. Int J Technol Assess Health Care. (2022) 38(1):E52. 10.1017/S0266462321001768 35959563

[4] Mukherjee K Walley T. Assessing the value of healthcare innovations: a proposal for an integrated health technology assessment–responsible innovation in health approach in the “new normal”. Int J Technol Assess Health Care. (2022) 38(1):E47. 10.1017/S0266462322000319 35549786

[5] The U.S. President's Emergency Plan for AIDS Relief (PEPFAR). Available online at: https://www.state.gov/pepfar/https://www.hiv.gov/federal-response/pepfar-global-aids/pepfar (accessed July, 2022).

[6] Pai M. How Many Variants And Deaths Are We Willing To Accept, Before We Protect The Whole World? (Jul 12, 2022). Available online at: https://www.forbes.com/sites/madhukarpai/2022/07/12/how-many-variants-and-deaths-are-we-willing-to-accept-before-we-protect-the-whole-world/?sh=75f5575f2233 (accessed July 14, 2022).

[7] Levin AT Owusu-Boaitey N Pugh S Fosdick BK Zwi AB Malani A Assessing the burden of COVID-19 in developing countries: systematic review, meta-analysis and public policy implications. BMJ Glob Health. (2022) 7(5):e008477. 10.1136/bmjgh-2022-008477 35618305 PMC9136695

[8] Erfani P Binagwaho A Jalloh MJ Yunus M Farmer P Kerry V. Intellectual property waiver for COVID-19 vaccines will advance global health equity. Br Med J. (2021) 374:n1837. 10.1136/bmj.n1837 34344728

[9] Neidleman J. The Social contract theory in a global context. (2022). Available online at: https://www.e-ir.info/2012/10/09/the-social-contract-theory-in-a-global-context/ (accessed June, 2022).

[10] African Development Bank Group. African Development Bank's Board approves landmark institution: Establishment of African Pharmaceutical Technology Foundation to transform Africa's pharmaceutical industry. Cote d'Ivoire: African Development Bank Group, News and Events (2022). Available online at: https://www.afdb.org/en/news-and-events/press-releases/african-development-banks-board-approves-landmark-institution-establishment-african-pharmaceutical-technology-foundation-transform-africas-pharmaceutical-industry-52727 (accessed July 16, 2022).

[11] The Africa Centres for Disease Control and Prevention (CDC). Available online at: https://africacdc.org/ (accessed August, 2022).

[12] Perehudoff K ‘t Hoen E Mara K Balasubramaniam T Abbott F Baker B A pandemic treaty for equitable global access to medical countermeasures: seven recommendations for sharing intellectual property, know-how and technology. BMJ Global Health. (2022) 7:e009709. 10.1136/bmjgh-2022-009709 35840169 PMC9295647

[13] Maxmen A. Unseating big pharma: the radical plan for vaccine equity. Nature. (2022) 606:226–33. Available online at: https://media.nature.com/original/magazine-assets/d41586-022-01898-3/d41586-022-01898-3.pdf (accessed July 26, 2022) 10.1038/d41586-022-01898-3 35831606

[14] Treaty for the establishment of the African Medicines Agency. (February 11, 2019). Available online at: https://au.int/sites/default/files/treaties/36892-treaty-0069_-_ama_treaty_e.pdf (accessed February 2022).

[15] Jerving S. Rwanda chosen to host the African Medicines Agency. (July 18, 2022). Available online at: https://www.devex.com/news/rwanda-chosen-to-host-the-african-medicines-agency-103653 (Accessed July 23, 2022).

[16] Mukanga D. Progress toward African Medicines Agency and vaccine manufacturing in Africa. DIA Global Forum. (July 2022). Available online at: https://globalforum.diaglobal.org/issue/july-2022/progress-toward-african-medicines-agency-and-vaccine-manufacturing-in-africa/ (accessed July 23, 2022).

[17] Marcus JL Killelea A Krakower DS. Preventative treatments may also not be available because of the high costs: perspective. Perverse incentives — hIV prevention and the 340B drug pricing program. N Engl J Med. (2022) 386:2064–6. 10.1056/NEJMp2200601 35621521

[18] Silva HP Lehoux P Miller FA Denis JL. Introducing responsible innovation in health: a policy-oriented framework. Health Res Policy Syst. (2018) 16:90. 10.1186/s12961-018-0362-5 30200985 PMC6131953

[19] WHO European Region technical reports. (August 2022). Available online at: https://www.who.int/europe/initiatives/the-oslo-medicines-initiative/technical-reports (accessed August 31, 2022).

[20] Benavides X. Inequitable by design: the law and politics of global COVID-19 vaccine access - and a way out. University of Michigan Journal of Law Reform. (2022) 56(2):93. 10.2139/ssrn.4104649

[21] United Nations Development Programme (UNDP) Global Dashboard. John Hopkins University UNDP Global dashboard for vaccine equity. Available online at: https://data.undp.org/vaccine-equity/ (accessed May, 2022).

[22] Stiglitz JE. Vaccinating the world against COVID-19 is a no-brainer. PLOS Glob Public Health. (2022) 2(5):e0000427. 10.1371/journal.pgph.0000427 PMC10021259 36962212

[23] Guglielmi G. COVID was twice as deadly in poorer countries. Nature. (2022) 10.1038/d41586-022-01767-z. [Epub ahead of print] 35764825

[24] John Hopkins University of Medicine Coronavirus Resource Center/COVID-19 Dashboard of Global Cases by the Center for Systems Science and Engineering (CSSE) at Johns Hopkins University. Available at: https://coronavirus.jhu.edu/map.html (accessed May, 2022).

[25] Akondeng C Njamnshi WY Mandi HE Agbor VN Bain LE Njamnshi AK. Community engagement in research in sub-saharan Africa: approaches, barriers, facilitators, ethical considerations and the role of gender—a systematic review protocol. BMJ Open. (2022) 12(5):e057922. 10.1136/bmjopen-2021-057922 35545398 PMC9096545

[26] Mehndiratta A Rajsekar K Singh M Tyagi K Sohail A Guzman J. How to build trusted priority-setting systems to increase value for money in health care decisions. Center for Global Development Blog Post. (September 14, 2022). Available online at: https://www.cgdev.org/blog/how-build-trusted-priority-setting-systems-increase-value-money-health-care-decisions (accessed September 21, 2022).

[27] Sehmi K Wale JL. Where national medicines policies have taken us with patient involvement and health technology assessment in Africa. Front Med Technol. (2022) 4:810456. 10.3389/fmedt.2022.810456 35281672 PMC8915114

[28] Jatoi I Sung H Jemal A. The emergence of the racial disparity in U.S. Breast-cancer mortality. N Engl J Med. (2022) 386(25):2349–52. 10.1056/NEJMp2200244 35713541

[29] https://www.who.int/publications/i/item/9789240027794.

[30] World Health Organization Global Benchmarking Tool. (2022). Available online at: https://www.who.int/tools/global-benchmarking-tools (accessed May 16, 2022).

[31] Wale J Scott A Hofmann B Garner S Low E Sansom L. Why patients should be involved in health technology assessment. Int J Technol Assess Health Care. (2017) 33(1):1–4. 10.1017/S0266462317000241 28528585

[32] Wale JL Chandler D Collyar D Hamerlijnck D Saldana R Pemberton-Whitely Z. Can we afford to exclude patients throughout health technology assessment? Front Med Technol. (2022) 3:796344. 10.3389/fmedt.2021.796344 35146487 PMC8821945

[33] Oortwijn W Jansen M Baltussen R. Evidence-Informed deliberative processes for health benefit package design - part II: a practical guide. Int J Health Policy Manag. (2021) 11(10):2327–36. 10.34172/ijhpm.2021.159 PMC9808268 34923809

[34] Ramponi F Twea P Chilima B Nkhoma D Chiumia IK Manthalu G Assessing the potential of HTA to inform resource allocation decisions in low-income settings: the case of Malawi. Front. Public Health. (2022) 10:1010702. 10.3389/fpubh.2022.1010702 36388387 PMC9650047

[35] World Health Organization (WHO) Health Innovation Group. Available online at: https://www.who.int/life-course/about/who-health-innovation-group/en/ (accessed May 17, 2022).

[36] Transforming our World: The 2030 Agenda for Sustainable Development. The United Nations 2030 Agenda for Sustainable Development and Universal Health Coverage. UN General Assembly. (October 21, 2015). UN Doc. A/RES/70/1. Available online at: https://sdgs.un.org/2030agenda (accessed October 25, 2021).

[37] https://www.who.int/publications/i/item/WHO-MHP-HPS-EML-2021.01.

[38] Vlad I. Establishing health technology assessment (HTA) in middle-income countries: a comparative analysis of the path towards institutionalisation in Thailand and the Philippines. (Ph.D. thesis), London School of Hygiene & Tropical Medicine, London (2020). Available online at: https://researchonline.lshtm.ac.uk/id/eprint/4659333/ (accessed June 2, 2021). 10.17037/PUBS.04659333

[39] The International Alliance for Patients’ Organizations (IAPO). Available online at: https://www.iapo.org.uk/ (accessed May, 2022).

[40] The Max Foundation. Available online at: https://themaxfoundation.org/about/ (accessed August 2022).

[41] Access to Oncology Medicines (ATOM) Coalition. Available online at: https://www.uicc.org/who-we-work/networks/access-oncology-medicines-atom-coalition (accessed June 16, 2022).

[42] The Medicines Patent Pool (MPP), a United Nations-backed public health organisation working to increase access to, and facilitate the development of life-saving medicines for low and middle income countries through voluntary licensing and patent pooling. Available online at: https://medicinespatentpool.org/ (accessed May 2022).

[43] Rumbold B Baker R Ferraz O Hawkes S Krubiner C Littlejohns P Universal health coverage, priority setting, and the human right to health. Lancet. (2017) 390(10095):712–4. 10.1016/S0140-6736(17)30931-5 28456508 PMC6728156

[44] UN Rare Diseases. On the 16th of December 2021, Resolution on ‘Addressing the challenges of persons living with a rare disease and their families’ (December 16, 2021). Available online at: https://www.globenewswire.com/news-release/2021/12/16/2354050/0/en/First-ever-United-Nations-Resolution-to-Increase-Visibility-for-the-300-Million-Persons-Living-with-a-Rare-Disease.html (accessed May 25, 2022).

[45] Wale JL Thomas S Hamerlijnck D Hollander R. Patients and public are important stakeholders in health technology assessment but the level of involvement is low - a call to action. Res Involv Engagem. (2021) 7(1):1. 10.1186/s40900-020-00248-9 33402216 PMC7783693

[46] Mukherjee K. Relevance of the newly defined health technology assessment: COVID-19 and beyond. Int J Technol Assess Health Care. (2021) 37:e44. 10.1017/S0266462321000192 33750491

[47] Kingkaew P Budtarad N Khuntha S Barlow E Morton A Isaranuwatchai W A model-based study to estimate the health and economic impact of health technology assessment in Thailand. Int J Technol Assess Health Care. (2022) 38(1):E45. 10.1017/S0266462322000277 35506420

[48] McGurn S. Changing Landscape of Evidence Charting New Assessment Pathways Q&A with Canadian Agency for Drugs and Technologies in Health (CADTH) CEO Suzanne McGurn. (2021). Available online at: https://globalforum.diaglobal.org/issue/october-2021/changing-landscape-of-evidence-charting-new-assessment-pathways/ (accessed October 2021).

[49] Javier H Guzman Cruz, HTAi2022 Plenary Three. Running Around in Circles; Time for Real Collaboration between Regulators, HTA Bodies and Clinicians. (June 29, 2022). Program available online at: https://www.htai2022.org/plenaries (accessed June 29, 2022).

[50] Javier Guzman. HTA in Question. Event 5. HTA in Low and Middle Income Countries. Moderator Pilar Pinilla-Domiguez NICE International. Consilium Scientific. (June 2, 2022). Available online at: https://www.youtube.com/watch?v=jBxx2_t_UBw (accessed June 2, 2022).

[51] Xie RZ Malik ED Linthicum MT Bright JL. Putting stakeholder engagement at the center of health economic modeling for health technology assessment in the United States. Pharmacoeconomics. (2021) 39(6):631–8. 10.1007/s40273-021-01036-3 33982198 PMC8166701

[52] Arntsen K Blount L Dickerson B Koola C Venable Y Wildman P. Patient-centered health technology assessment: a perspective on engagement in health technology assessment by three patient organizations and a health technology assessment body. Int J Technol Assess Health Care. (2022) 38(1):E76. 10.1017/S0266462322000587 36263665

[53] Spangler J Huth T Xie R. Letter to editor: patient perspectives: an integral part of health technology assessment methodology. Int J Technol Assess Health Care. (2022) 38(1):E85. 10.1017/S0266462322003270 36524546

[54] Modi ND Abuhelwa AY McKinnon RA Boddy AV Haseloff M Wiese MD Audit of data sharing by pharmaceutical companies for anticancer medicines approved by the US food and drug administration. JAMA Oncol. (2022) 8(9):1310–6. 10.1001/jamaoncol.2022.2867 35900732 PMC9335250

[55] Van Zimmeren E Minssen T Paemen L Van Dyck W Luyten J Janssens R Barbier L Simoens S Pouppez C Cleemput I Vinck I. Compulsory licensing for expensive medicines. Health Services Research (HSR). Brussels. Belgian Health Care Knowledge Centre (KCE). KCE Reports. (2022) 356. D/2022/10.273/35. Available online at: https://kce.fgov.be/en/compulsory-licensing-for-expensive-medicines (accessed June 16, 2022).

[56] Greenhalgh T Fahy N Shaw S. The bright elusive butterfly of value in health technology development comment on “providing value to new health technology: the early contribution of entrepreneurs, investors, and regulatory agencies”. Int J Health Policy Manag. (2018) 7(1):81–5. 10.15171/ijhpm.2017.65 29325407 PMC5745872

[57] Lehoux P Miller FA Daudelin G Denis JL. Why learning how to chase butterflies matters: a response to recent commentaries. Int J Health Policy Manag. (2018) 7(3):286–7. 10.15171/ijhpm.2017.114 29524961 PMC5890077

[59] https://fairpharmascorecard.org/.

[60] Athanasiou D Bakker A Brooke N Carr M Chlebus M Gorman S Spooner S. DIA Europe 2022. Patient Engagement Action Plan. Seven Steps to Move the Needle (2022). An interactive session at DIA Europe 2022, co-hosted by the European Federation of Pharmaceutical Industries and Associations and the European Patients’ Forum. Available online at: https://globalforum.diaglobal.org/issue/july-2022/patient-engagement-action-plan/ (accessed September 4, 2022).

[61] Patient involvement in the development, regulation and safe use of medicines. CIOMS Working Group report. Geneva, Switzerland: Council for International Organizations of Medical Sciences (CIOMS), 2022. Available online at: https://cioms.ch/publications/product/patient-involvement/ (accessed September 4, 2022). 10.56759/iiew8982

[62] Press release, GSK planning to invest a billion UK pounds, in decreasing infectious diseases (Date). Available online at: https://www.gsk.com/en-gb/media/press-releases/gsk-announces-1-billion-rd-investment-over-ten-years-to-get-ahead-of-infectious-diseases-in-lower-income-countries/ (accessed July, 2022).

[63] Press release, Sanofi to provide 30 essential drugs on non-profit basis in low-income countries (July 4, 2022). Available online at: https://firstwordpharma.com/story/5607271 Ref: GlobeNewswire, Financial Post, Finanz Nachrichten. Matthew Dennis (accessed July, 2022).

[64] Press release, Pfizer to provide all patented drugs, vaccines to poor countries on no-profit basis (May 25, 2022). Available online at: https://firstwordpharma.com/story/5579429 Ref: Pfizer, ABC News, Fidelity, MarketWatch, The Wall Street Journal, BBC News, The Guardian. Matthew Dennis (accessed July, 2022).

[65] Oortwijn W Husereau D Abelson J Barasa E Bayani DD Santos VC Designing and implementing deliberative processes for health technology assessment: a good practices report of a joint HTAi/ISPOR task force. Int J Technol Assess Health Care. (2022) 38(1):e37. 10.1017/S0266462322000198 35656641 PMC7613549

[66] Murphy A Bere N Vamvakas S Mavris M. The added value of patient engagement in early dialogue at EMA: scientific advice as a case study. Front Med (Lausanne). (2022) 8:811855. 10.3389/fmed.2021.811855 35127766 PMC8811124

[67] Aleman A Galan A P. Impact of health technology assessment in litigation concerning access to high-cost drugs. Int J Technol Assess Health Care. (2017) 33(4):411–4. 10.1017/S0266462317000575 28756785

[68] African Medicines Agency Treaty Alliance (AMATA) Joint Statement by AMATA welcoming the African Medicines Agency coming into force (2021). Available online at: https://www.iapo.org.uk/news/2021/nov/4/joint-statement-amata-welcoming-african-medicines-agency-coming-force (accessed July 16, 2022).

[69] World Patients Alliance (WPA). Available online at: www.worldpatientsalliance.org (accessed September, 2022).

